# Regionalized Pathology Correlates with Augmentation of mtDNA Copy Numbers in a Patient with Myoclonic Epilepsy with Ragged-Red Fibers (MERRF-Syndrome)

**DOI:** 10.1371/journal.pone.0013513

**Published:** 2010-10-20

**Authors:** Anja Brinckmann, Claudia Weiss, Friederike Wilbert, Arpad von Moers, Angelika Zwirner, Gisela Stoltenburg-Didinger, Ekkehard Wilichowski, Markus Schuelke

**Affiliations:** 1 Department of Neuropediatrics, Charité University Medical School, Berlin, Germany; 2 DRK-Kliniken Westend, Berlin, Germany; 3 Department of Neuropathology, Charité University Medical School, Berlin, Germany; 4 Department of Pediatrics and Pediatric Neurology, Georg August University, Göttingen, Germany; 5 NeuroCure Clinical Research Center, Charité University Medical School, Berlin, Germany; Stanford University School of Medicine, United States of America

## Abstract

Human patients with myoclonic epilepsy with ragged-red fibers (MERRF) suffer from regionalized pathology caused by a mutation in the mitochondrial DNA (m.8344A→G). In MERRF-syndrome brain and skeletal muscles are predominantly affected, despite mtDNA being present in any tissue. In the past such tissue-specificity could not be explained by varying mtDNA mutation loads. In search for a region-specific pathology in human individuals we determined the mtDNA/nDNA ratios along with the mutation loads in 43 different *post mortem* tissue samples of a 16-year-old female MERRF patient and in four previously healthy victims of motor vehicle accidents. In brain and muscle we further determined the quantity of mitochondrial proteins (COX subunits II and IV), transcription factors (NRF1 and TFAM), and VDAC1 (Porin) as a marker for the mitochondrial mass. In the patient the mutation loads varied merely between 89–100%. However, mtDNA copy numbers were increased 3–7 fold in predominantly affected brain areas (e.g. hippocampus, cortex and putamen) and in skeletal muscle. Similar increases were absent in unaffected tissues (e.g. heart, lung, kidney, liver, and gastrointestinal organs). Such mtDNA copy number increase was not paralleled by an augmentation of mitochondrial mass in some investigated tissues, predominantly in the most affected tissue regions of the brain. We thus conclude that “futile” stimulation of mtDNA replication *per se* or a secondary failure to increase the mitochondrial mass may contribute to the regionalized pathology seen in MERRF-syndrome.

## Introduction

Mutations of the mitochondrial DNA (mtDNA) cause a variety of serious genetic disorders with maternal inheritance [1]. One example is the MERRF-syndrome (myoclonic epilepsy with ragged-red fibers, MIM 545000), which is characterized by myoclonic seizures, ataxia, dementia, muscle weakness and accumulations of structurally abnormal mitochondria in the skeletal muscle [2]. MERRF-syndrome is most frequently associated with the m.8344A→G mutation in the mitochondrial tRNA^Lys^ gene (*MTTK*) [3]. The mutation usually occurs in heteroplasmic state where wildtype and mutant mtDNA copies coexist. Generally, MERRF patients have a high percentage of mutant mtDNA (>90%) in blood and muscle and the degree of heteroplasmy ( = mutation load) is evenly distributed between different organs [4]–[6], which stands in contrast to the MELAS-syndrome (MIM 540000), where more variation is found [7]. Cultured myotubes from MERRF patients show a biochemical defect only if their mutation load surpasses 85% and show a steep decline of COX-activity and mitochondrial translation rate beyond this threshold [8]. Even though mitochondria are present in every cell, MERRF-syndrome has a regionalized pathology, predominantly involving the brain (hippocampus, cerebral cortex and striatum) and the skeletal muscle, but often sparing the heart and other intestinal organs. In the past, researchers have tried to explain this phenomenon by differences of mutation load for the m.8344A→G mutation in the various organs. However, various studies did not confirm such an association [4]–[6], [9]. Alternatively, it was hypothesized that the affected tissues had lower threshold levels for clinical manifestation than their unaffected counterparts. Other explanations were tissue specific mechanisms for the regulation of energy metabolism, potentially at the level of mtDNA replication, transcription or translation.

**Figure 1:**
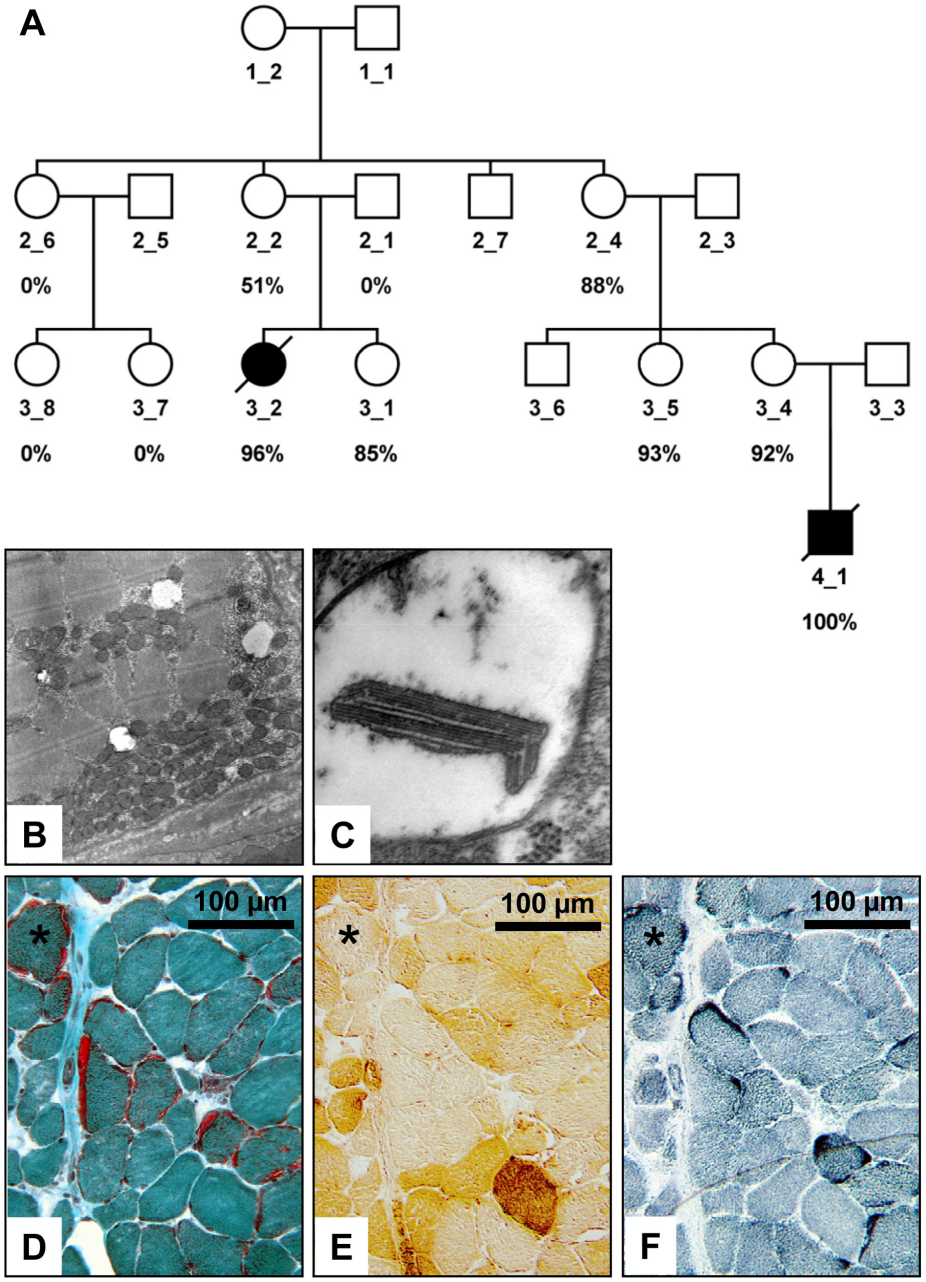
Analysis of the MERRF patient and her family members. (**A**) Pedigree of the family of the index patient (3_2). The mutation loads for the m.8344A→G mtDNA mutation, as determined by Pyrosequencing® are given below the pedigree symbols. The black symbols depict patients, who were clinically affected with MERRF-syndrome; however, even family members with 93% mutant mtDNA in blood cells did not report any MERRF-specific symptoms and their symbols thus remain white. (**B**) The electron microscopic image of the *iliopsoas* muscle (12,000×) shows massive subsarcolemmal accumulations of mitochondria, which represent the contents of a ragged-red fiber. (**C**) At higher magnification (85,000×) ballooned mitochondria with paracrystalline inclusions can be seen. (**D**) Ragged-red fibers in the Gömöri trichrome stain of the *iliopsoas* muscle that are COX negative in (**E**), and show subsarcolemmal accumulations of enzyme activity in the SDH stain (**F**).

Here we studied the regionalized pathology of MERRF-syndrome on tissue and molecular level through investigation of 43 *post mortem* tissue-samples from different regions of a 16-year-old girl with MERRF-syndrome and of blood DNA samples from her family (Figure 1A).

## Methods

### Ethics statement

Written informed consent according to the Declaration of Helsinki was obtained from the involved persons and guardians. The study on human subjects was approved by the IRB of the Charité. Consent for the animal studies was obtained from the LaGeSo Berlin (O 0083/08) according to the animal welfare act.

### Morphometric analysis

**Figure 2:**
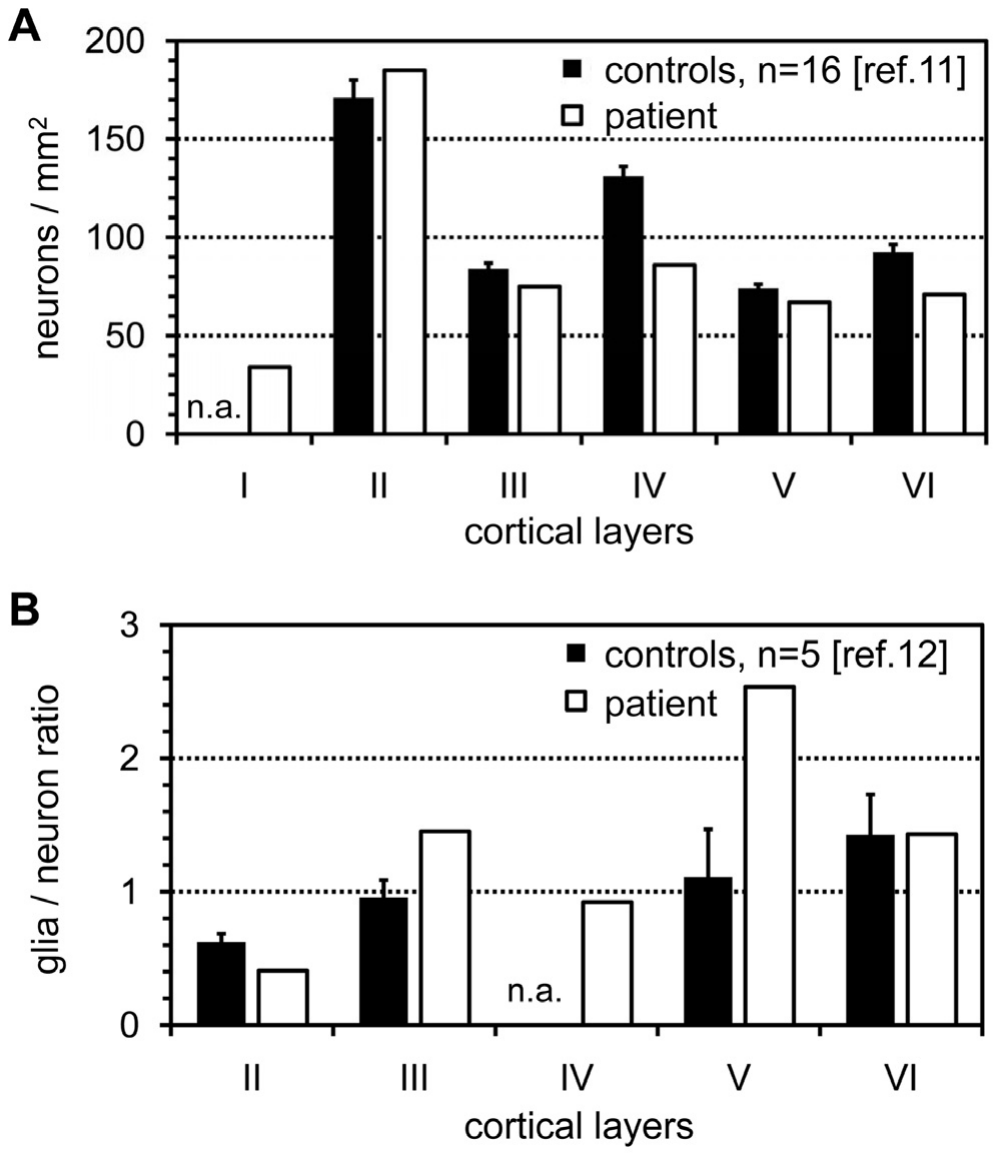
Morphometric analysis of neuronal cell density in the insular cortex of the index patient. (**A**) Density of cortical neurons using Abercrombie correction. The open bar from the patient represents the neuronal density from 130,000 µm^2^ of cross-sectional plane per cortical layer. (**B**) Ratio between the numbers of glial *versus* neuronal cells. Black bars, control individuals from the literature (reference provided); open bars, MERRF patient; the whiskers depict the SEM; n.a., data not available.

A tissue sample from the patient's insular cortex (Brodman area 43) including all neuronal layers and the white matter was fixed for >4 weeks in phosphate-buffered 4% formaldehyde, dehydrated and subsequently embedded in paraffin. Microtome sections of 5 µm were mounted on glass slides, deparaffinized, stained with anti-NeuN and subsequently with Cy3-labeled fluorescent secondary antibodies. The cell nuclei were counterstained with DAPI. An unbiased counting frame of 186.2×139.6 µm^2^ was then systematically moved through the cortical layers of interest and photographs were taken on the UV and Cy3-channel through a 20× oil immersion objective (Leica Microsystems, Wetzlar, Germany) with a SPOT3 cooled CCD camera (Visitron, Puchheim, Germany). From each cortical layer we analyzed five such frames which thus represented 130,000 µm^2^ of cross-sectional plane per respective cortical layer. The two images from the DAPI and the Cy3-channel were superimposed with false colors and on each frame we separately counted the total number of DAPI positive nuclei *versus* those nuclei that co-localized with a NeuN-signal representing neuronal cells that had been cut in the plane of the nucleus. All 2D-counts were corrected with the Abercrombie formula [10] taking into consideration the thickness of the section (5 µm) and the diameter of the cell nuclei. The Ferret's diameter of the nuclei was 14±5 µm and had been determined in 50 random neuronal nuclei at their largest optical cross-sectional diameter with a 63× oil immersion objective. The Abercrombie-corrected cell densities were then compared to published reference values that had been obtained by the same staining and 2D-counting method [11], [12]. The final results were then depicted as neurons/mm^2^ and as glia/neuron ratio (Figure 2A,B).

### Pyrosequencing® assay for determination of mutation load

DNA was isolated from blood cells and from tissues according to standard procedures using a 96-well tissue DNA extraction kit (Qiagen, Hilden, Germany). We amplified a 139 bp PCR-product of the mitochondrial tRNA^Lys^ gene with the primer pair 5′-CCC TAT AGC ACC CCC TCT AC-3′ and 5′-Biotin-TGG GCC ATA CGG TAG TAT TT-3′. The PCR product was sequenced using the automated PSQ™ HS 96A system with the primer 5′-TAA GTT AAA GAT TAA GAG A-3′. To correct for potential systematic errors of the assay, all data points of the samples were normalized to a calibration curve, generated with standardized mixtures of wildtype and mutant mtDNA (R^2^ = 0,9997; Figure S3).

### qPCR for determination of mtDNA copy numbers

The following primers and probes were used for amplification one gene encoded by the mtDNA and another single copy gene encoded by the nDNA: ***MTND1*** **( = mtDNA copy numbers):** (F) 5′-CTA CAA CCC TTC GCT GAC GC-3′, (R) 5′-ACG GCT AGG CTA GAG GTG GC-3′, (P) FAM-CCA TCA CCC TCT ACA TCA CCG CCC-X-TAMRA-3′ and for ***MMP1*** **( = nuclear DNA copy numbers):** (F) 5′-GCC AGG GTA CTG CAC TAG CAT G-3′, (R) 5′-GAG GCC CTA ACA TTC TCT GCA CT-3′, (P) FAM-TGT GCT ACA CGG ATA CCC CAA GGA CAT C-X-TAMRA-3′. The PCR for each sample was run in triplicate on an ABI PRISM 7700 sequence detection system (Applied Biosystems) with a hotstart Taq polymerase (Platinum Taq). A 10 min denaturation step at 94°C was followed by 45 cycles of denaturation at 94°C for 30 s and annealing/extension at 67°C for 60 s. The TaqMan readings were analyzed using the “Sequence Detector” v1.6.3 software (Applied Biosystems). On each 96-well plate we amplified in parallel a standard dilution series (10^0^–10^7^ copies/µl) for every gene, which subsequently enabled us to determine absolute copy numbers while taking into account the different PCR efficiencies [13]. If the standard deviation between the reads of a triplicate sample was above 10% the measurement had to be repeated.

### Western blot

Protein was extracted from tissue samples of the patient and the controls after homogenization in RIPA buffer with a proteinase inhibitor cocktail (Complete®, Roche-Diagnostics, Basel, Switzerland), 50 µg protein were separated through denaturating SDS-PAGE with the Laemmli system and blotted on nitrocellulose membranes by the semidry method (Biometra, Göttingen, Germany). The blots were first probed with antibodies against Porin (VDAC1), TFAM, NRF1, COX IV, COX II, β-Tubulin, and GAPDH and subsequently with corresponding peroxidase-labeled secondary antibodies. Bands were visualized by chemiluminescence. All antibodies used in this study are described in Table S2. β-Tubulin and GAPDH bands were used as loading controls for brain and muscle respectively.

## Results

### Case history

The girl was the second child of healthy non-consanguineous German parents. Her older sister is healthy. Pregnancy, birth and postnatal development were normal. Myoclonic-astatic seizures started at 4 years of age. Henceforward symptoms progressed to severe pharmaco-resistant myoclonic epilepsy with series of bitemporal spike-waves on EEG and several seizures daily, progressive ataxia, deafness (hearing threshold of 65 dB SPL), and mental retardation (HAWIK IQ = 76 at the age of 9 years). Serum lactate (4.1 mmol/l, N<2.0) and alanine (0.60 mmol/l, N<0.48) concentrations were increased and a diagnostic muscle biopsy at the age of 9 years revealed the presence of ragged-red and COX-negative fibers (Figure 1B–F). At 14 years of age echocardiography and ECG were normal and cranial MRI revealed *ex vacuo* dilatation of the lateral ventricles and hippocampal sclerosis. Two years later she was found lifeless in bed and could not be resuscitated. Autopsy at 48 h *post mortem*, during which we were allowed to take small specimens (Table S1), did not discover any cardiac or pulmonary abnormalities, excluded asphyxia or aspiration, and finally *status epilepticus* during sleep was suspected as the cause of her death. As controls we used specimens of the same tissues from four previously healthy women (16 to 32 years), who had suffered fatal motor vehicle accidents. Additionally, we analyzed the segregation of the mutation and its mutation load in blood samples of different family members (Figure 1A).

### Reduced neuronal density at the insular cerebral cortex

In our MERRF patient we found a reduction of neuronal density in the cortical layers IV, V, and VI of more than three times the SEM if compared to normal controls from the literature [11], [12]. The ratio between glial and neural cells (Figure 2B) was increased in the layers III and V (no reference data were available for cortical layer IV) suggesting a replacement of neurons by glial cells. However, the reduction in the number of cortical neurons was not as obvious as described in a review by Sparaco *et al.* (1993), which may be due to the much younger age of our patient in whom degenerative changes might just have begun [14].

### Little variation between mutation loads of different tissues

The m.8344A→G mutation loads in the blood of family members and in the tissue biopsy specimens were determined by a highly accurate Pyrosequencing® assay. This real-time sequencing technology allows quantification of two mitochondrial alleles that differ at one nucleotide position with down to 1% heteroplasmy [15], [16]. In the blood of the patient we measured a mutation load of 96%. As the mutation loads in blood cells from the clinically unaffected relatives ranged from 0–93% (Figure 1A), we assume a critical threshold level for clinical manifestation in this family between 93–96% for the m.8344A→G mutation. This result confirms other studies that found mutation loads >90% in blood or muscles of MERRF patients with clinical or histochemical muscle abnormalities [17]–[19]. In a severely affected nephew of our patient (individual 4_1, Figure 1A), who had died also from *status epilepticus* at the age of 2 years, we even detected a mutation load of 100% ( = homoplasmy) in blood cells. Such a high mutation load had not been reported before and had originally been considered incompatible with life because COX-activities were extremely low in cultured myotubes with homoplasmy for the mutation [8]. Next, we determined the mutation loads in the 43 tissue samples of the index patient with the same Pyrosequencing® assay. The mutation loads were more or less evenly distributed between 89–100% (Table S1), without preference for a specific tissue type. Only liver and pancreas had lower mutation loads of 67 and 73%, respectively. Similar uniform distributions were described before [4]–[6], failing to explain the regionalized pathology of MERRF-syndrome.

### mtDNA copy numbers are preferably increased in the most affected tissues

**Figure 3:**
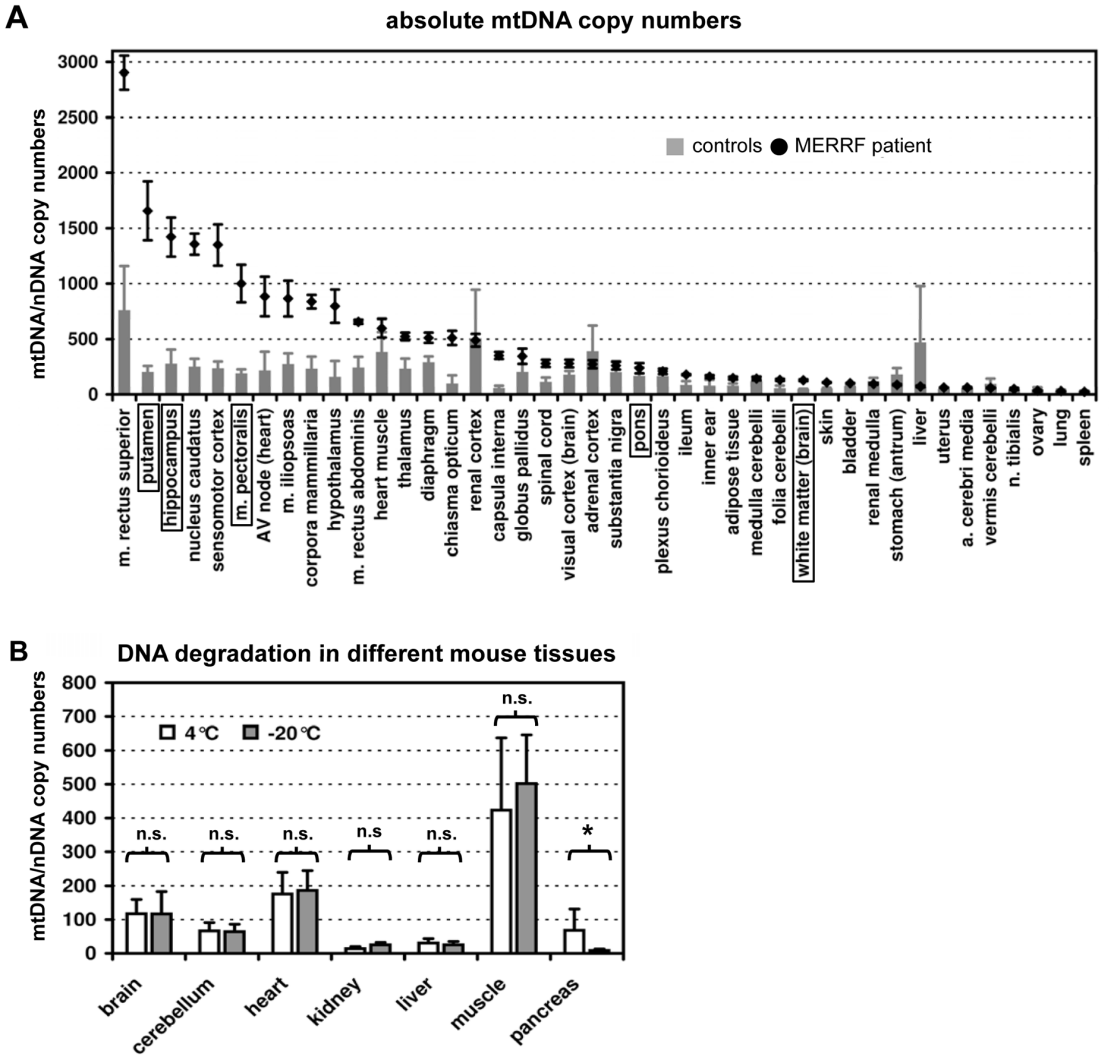
Determination of the absolute mtDNA copy numbers per cell in different tissues by quantitative real-time PCR (TaqMan®). (**A**) The ratio between mtDNA and nDNA copy numbers. The grey bars indicate mean values of the four controls (each sample analyzed in quadruplicate) and the black diamonds indicate mean values of the measurements from the patient's tissues. The whiskers represent the standard deviation; the tissues encircled by boxes were further investigated on the protein level. (**B**) Influence of the storage conditions on the mtDNA/nDNA ratios. With the exception of the pancreas, no significant differences on the 1% confidence level were found between the two storage conditions with regard to the mtDNA/nDNA ratios. n.s. not significant; * p<0.001. In pancreas the nuclear DNA degraded faster than the mtDNA at 4°C.

As cells from different tissues vary considerably in their mtDNA content, we next investigated whether the remaining wildtype mtDNA molecules per cell might correlate with the regionalized symptoms. In order to calculate this figure we determined the mtDNA copies per cell for all tissues by quantitative real-time PCR (TaqMan®) of the mitochondrial encoded *MTND1* gene (NADH:ubiquinone oxidoreductase, subunit ND1) *versus* the single copy nuclear *MMP1* gene (Matrix metalloproteinase 1). MtDNA copy numbers were increased between 3–7 fold in those tissues of the patient that were predominantly affected by the MERRF-mutation (hippocampus, cortex, striatum and skeletal muscle), but not in other tissues such as heart, lung, liver or intestinal organs (Figure 3A). Using the mtDNA copy numbers and the mutation load for each tissue we calculated the number of residual wildtype mtDNA copies per cell. There, the highly affected tissues still had the highest remaining absolute numbers of wildtype mtDNA copies per cell (Figures S1 and S2).

### Effect of the storage conditions on the ratio between mtDNA and DNA

As the ratio between mtDNA and nDNA copy numbers could strongly be influenced by unequal degradation of mitochondrial *versus* nuclear DNA during storage of the body in the morgue at 4°C [20], we investigated mice carcasses which had been kept under various storage conditions. We measured the mtDNA/nDNA ratios in 7 representative mouse tissues (brain, cerebellum, heart, kidney, liver, muscle and pancreas) under two storage conditions. The qPCR experiment was performed in triplicate as previously described [21]. After atlanto-axial dislocation the animals were kept at room temperature for 6 h and then organs were removed and stored for 60 h at 4°C *versus* immediately at −20°C. With the exception of pancreas (p<0.001) we did not find significant differences in mtDNA/nDNA ratios (Figure 3B). Only the nuclear DNA of the pancreas seemed to be more prone to degradation at 4°C as compared to −20°C. These results confirmed the constancy of the mtDNA/nDNA ratio over the selected time period, especially in brain and muscle. Hence we have reason to assume that the mtDNA/nDNA ratios measured with the TaqMan®assay truly reflect those at the time of death, with the exception of the pancreas.

### Determination of the mitochondrial mass

**Figure 4:**
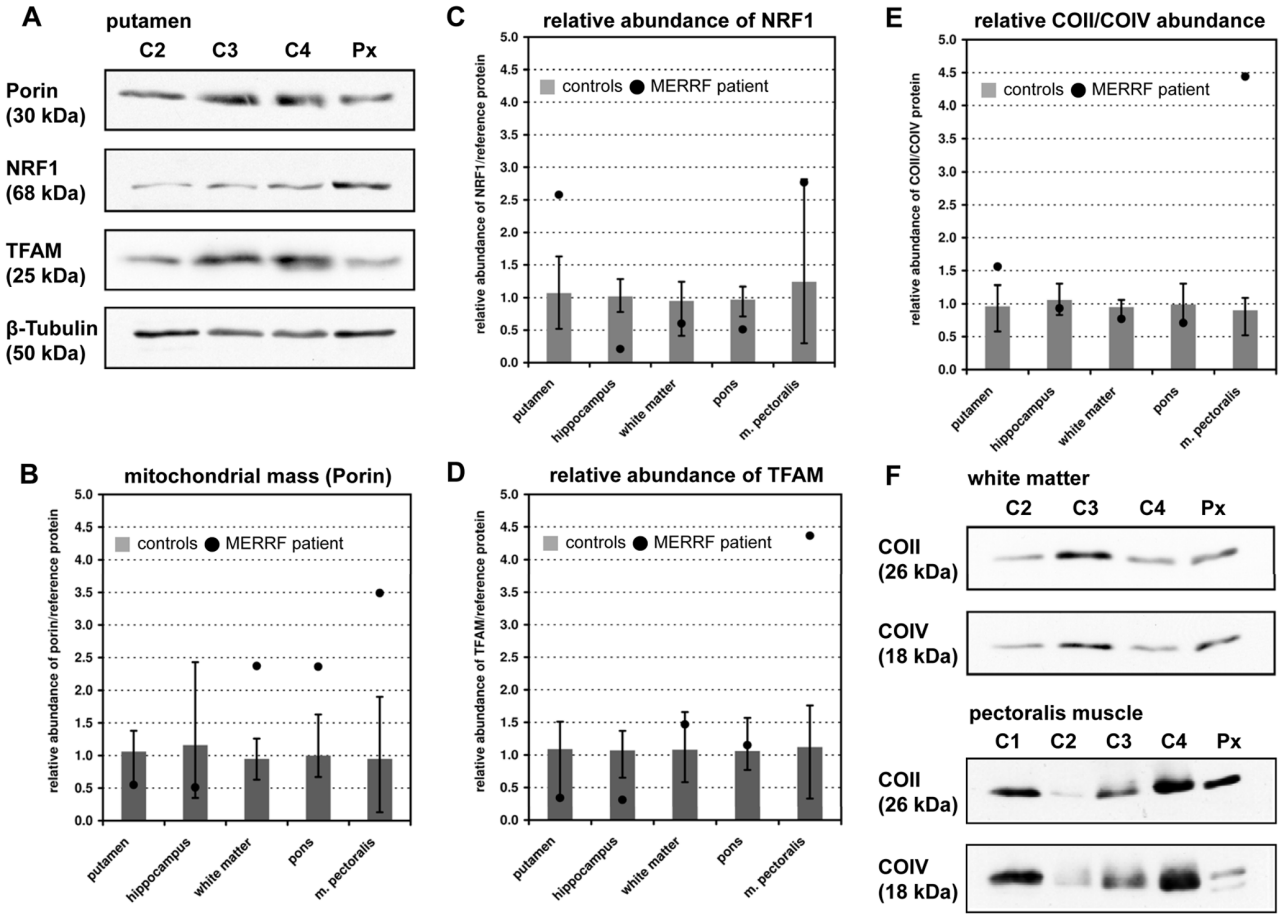
Determination of protein abundance for structural mitochondrial proteins and for transcription factors. (**A**) Western blot of tissue samples from the putamen of three controls (C2-4) and the patient (individual 3_2 on Figure 1A). Protein abundance was determined semi-quantitatively through densitometry. β-Tubulin was used as loading control. (**B**) Results of the semi-quantitative analysis of the mitochondrial mass as represented by anti-Porin (VDAC1) immune reactivity. The whiskers represent the absolute (100%) range of all three control measurements. The result of the patient is represented as a black dot. Figures (**C**) and (**D**) represent the quantification of the transcription factors NRF1 and TFAM. (**E**) Relative abundance of the nuclear encoded subunit of cytochrome C oxidase (COIV) *versus* an mtDNA-encoded subunit (COII) of the same complex. The immune staining with both antibodies was done on the identical blot which secured the comparability. (**F**) Only in muscle the mtDNA-encoded COII subunit was much higher expressed (4.5×) than its nDNA encoded counterpart. ***Note***: Due to the lack of sufficient autopsy material the loading of each lane could not be adjusted. However, these Western blots are only intended to illustrate an equal abundance of proteins encoded by the nuclear (COIV) *versus* the mtDNA (COII) and the absence of this balance in the muscle of the MERRF patient.

Further, we investigated whether the elevated mtDNA/nDNA ratios were solely due to enhanced mtDNA-replication or caused by a real increase of the number of mitochondrial organelles per cell ( = mitochondrial mass). We thus extracted total protein from brain specimens and pectoralis muscle of patient and controls and performed a semi-quantitative Western blot analysis using antibodies against Porin (VDAC1). This protein being highly expressed at the outer mitochondrial membrane [22] serves as a marker for mitochondrial mass [23]. Due to the small volumes of many biopsy specimens, we were limited in the number of tissues to be analyzed by this method but we were able to investigate hippocampus, putamen, and pectoralis muscle, representing tissues that are affected in MERRF-syndrome, and the occipital white matter and the pons, representing tissues that remain mostly unaffected. The mitochondrial mass of the patient's hippocampus and putamen was within the range of controls (Figure 4B). In contrast, a more than two-fold increase was found in pons, white matter, and skeletal muscle. Thus, we did not find a general correlation between increased mtDNA abundance and increased mitochondrial mass. On the contrary, in the investigated brain regions we **either** found an increase in mtDNA copy numbers **or** in mitochondrial mass. Only in skeletal muscle we found a parallel increase of both parameters.

Next we examined, whether high mtDNA copy numbers might be secondary to a stimulation of mtDNA transcription *via* up-regulation of mitochondrial transcription factors. Semi-quantitative Western blot analysis of the transcription factors NRF1 (nuclear respiratory factor 1) acting on nuclear genes that encode respiratory chain subunits, and TFAM (mitochondrial transcription factor A) stimulating mtDNA transcription, revealed no correlation of their amounts with the mtDNA copy number of the analyzed brain regions (Figure 4C,D). The TFAM levels in the organ with one of the largest increase in mtDNA copy numbers (putamen) were even the lowest. The situation looked different in the patient's pectoralis muscle where a fourfold up-regulation of TFAM was paralleled by a similar increase of mitochondrial mass.

Finally we investigated the balance of abundance between proteins encoded by the mitochondrial *versus* the nuclear genome. For that we determined the integrated band densities on Western blots that had been co-stained with antibodies against COX subunit II (26 kDa, mtDNA encoded) and COX subunit IV (18 kDa, nDNA encoded). Here again, the protein expression was balanced in all investigated brain regions irrespective of their mtDNA copy number and mitochondrial mass (Figure 1E). Only in muscle we found this relation considerably and unexpectedly skewed more than fourfold towards the mtDNA encoded subunit Cox II (Figure 4E,F). As mitochondrial mass was increased in a similar range in muscle (3.5 fold) we hypothesize that synthesis and import of nuclear encoded mitochondrial proteins might not have been increased strictly in parallel. The subsequent deposition of overproduced subunits might have led to the appearance of paracrystalline inclusions in many of the muscle mitochondria as seen on Figure 1C. As alternative explanation, the intense Cox II band might not represent intact subunits but misfolded or partially miscomposed polypeptide chains caused by a lack of tRNA^Lys^ which recognizes 4 of 227 codons in Cox II mRNA.

## Discussion

It is generally agreed that ATP deficiency and increased production of reactive oxygen species (ROS) are crucial factors in the pathophysiology of mitochondrial disorders due to mtDNA mutations and deletions [1], [24]. ROS are generated through electron leaks in a dysfunctional respiratory chain, however, their harmful effects on the mtDNA may be mitigated, at least partially, by compensatory up-regulation of mtDNA copy numbers and mitochondrial mass. H_2_O_2_ in the low µM range increases mtDNA copy numbers and mitochondrial mass in cultures of yeast as well as human cells and cybrids harboring the 4,977 bp deletion [25], [26]. Moreno-Loshuertos *et al.* (2006) even went as far as to propose that respiration deficient phenotypes caused by certain mouse mtDNA haplotypes on a uniform cybrid background be compensated *via* ROS-mediated increase of mtDNA copy numbers [27], a view that did not remain unchallenged [28]. Hori et al. (2009) proposed an attractive link between increased ROS production and enhanced mtDNA replication *via* increased nicking by Ntg1 of oxidized single-stranded mtDNA at the origin of replication and thus initiating rolling cycle replication of yeast mtDNA [29]. Whether a homologous mechanism applies for mammalian mtDNA is currently unknown.

**Figure 5:**
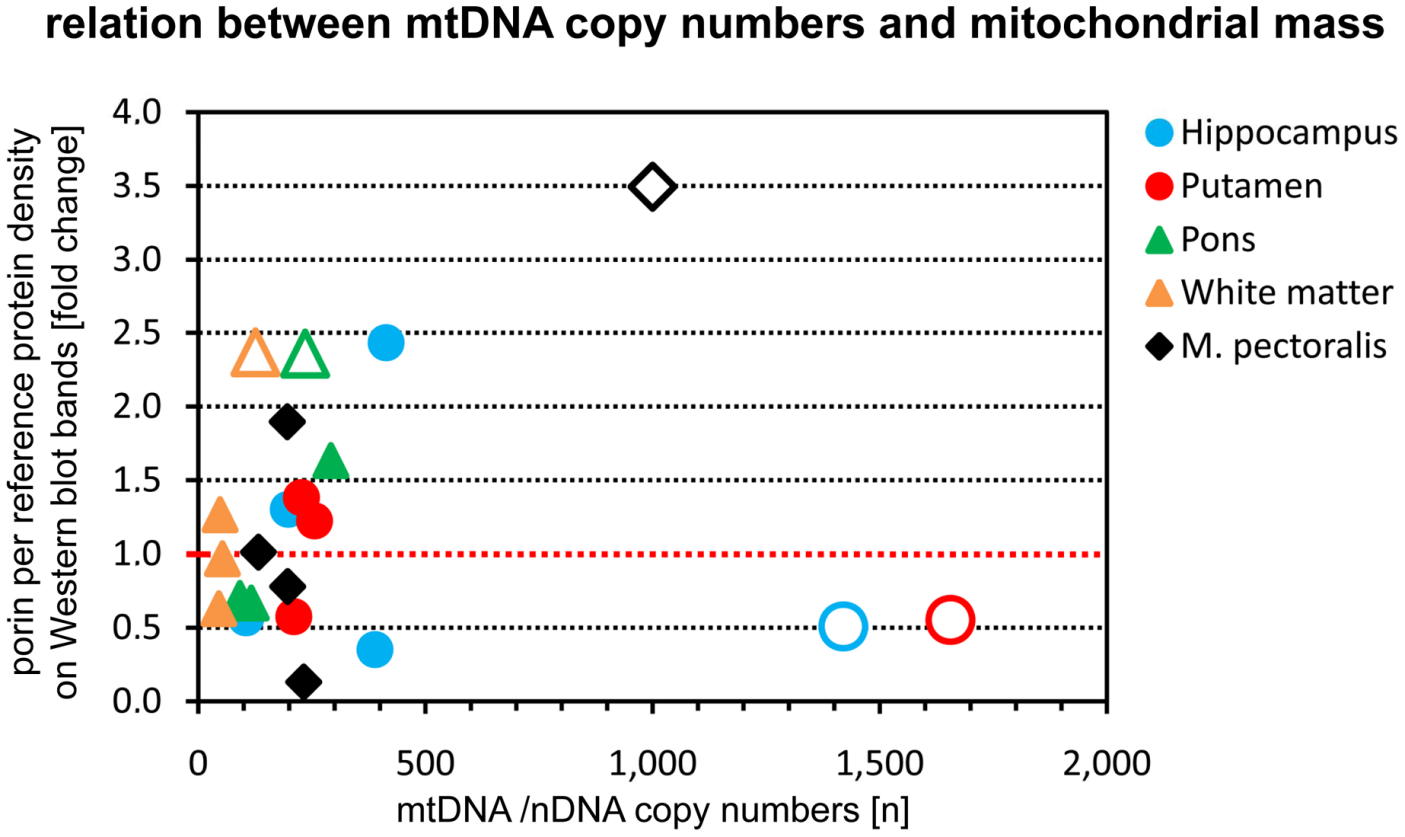
Relation between mtDNA copy numbers and mitochondrial mass. The figure depicts an X-Y plot of mtDNA copy numbers *versus* mitochondrial mass in five representative tissues. Mitochondrial mass is represented by the reference-corrected density of Western blot bands from the outer mitochondrial membrane protein porin. The red line depicts the average of the mitochondrial mass, which was calculated as the geometric means from all control individuals. Circles represent clinically affected tissues and triangles unaffected tissues. Closed symbols represent the control individuals and open symbols the patient. Muscle, which was clinically only mildly affected, is represented by the diamond shaped symbol.

Here we demonstrate for the first time that the augmentation of mtDNA copy numbers in a MERRF patient is a tissue-specific phenomenon, which might help to explain the preponderance of certain organs for disease-specific symptoms. However, the question remains whether the up-regulation of mtDNA copy numbers is a compensatory, albeit insufficient step caused by increased ROS production in metabolically “demanding” tissues or whether such an up-regulation, especially if not followed by a subsequent increase in mitochondrial mass, may be harmful *per se* as it may exhaust the resources of the mitochondria. As TFAM is known to have an influence mtDNA copy numbers [24], we investigated its expression in predominantly affected and unaffected tissues. In brain we found an especially low expression for putamen and hippocampus which did not up-regulate their mitochondrial mass despite high mtDNA copy numbers (Figure 5). This stood in contrast to white matter and pons that augmented their mitochondrial mass. In the muscle of the patient, elevated mitochondrial mass and TFAM abundance went strictly parallel demonstrating that the mechanism(s) to increase mitochondrial mass are at least partially independent of TFAM. This has already been shown in muscle specific *Tfam*-knockout mice that were able to up-regulate mitochondrial mass in the absence of Tfam [30].

Unfortunately, lack of sufficient autopsy material prohibited the investigation of other tissues. We are aware that the data being obtained from a single individual have to be confirmed by further investigations of MERRF patients and preferably also in individuals with other mtDNA mutations, especially the MELAS (m.3243A→G) mutation, to explore whether a tissue-specific increase in mtDNA copy numbers is a MERRF-specific phenomenon or whether it may be generalized for other mitochondrial disorders as well.

## Supporting Information

Figure S1 Increase [in %] of the mtDNA copy numbers in the patient in relation to the mean of the four controls. The tissues mainly affected by the MERRF-syndrome cluster on the left side of the chart.
(0.65 MB TIF)

Figure S2 Absolute numbers of residual wildtype mtDNA molecules per cell in the patient tissues (magenta dots). The blue bars depict the mean and the whiskers the standard deviation of the mtDNA copy numbers in the four controls.
(1.01 MB TIF)

Figure S3 Calibration curve for the Pyrosequencing assay. Measurements from the assay were plotted against the expected degrees of heteroplasmy from known mixtures between plasmid preparations containing the wildtype and mutant DNA sequence. All measurements were performed in triplicate and the standard deviation is indicated by whiskers. Due to the high precision of the method with small standard deviations, not all the whiskers can be seen. The regression line forms a smooth curve with the above mentioned equation and a very good approximation of the measurements. All raw measurements of the samples were normalized to the regression curve.
(0.33 MB TIF)

Table S1 Mutation loads ( = degrees of heteroplasmy in [%]) of all the organs as measured by the Pyrosequencing assay. The numbers indicate the average of three measurements ± standard deviation.
(0.95 MB TIF)

Table S2 Primary antibodies and their dilutions used for Western blot and immunohistochemistry.
(0.11 MB TIF)
