# Supplementary figures and images for: Regionalized Pathology Correlates with Augmentation of mtDNA Copy Numbers in a Patient with Myoclonic Epilepsy with Ragged-Red Fibers (MERRF-Syndrome)
Source: PLoS One. 2010 Oct 20;5(10):e13513. doi: 10.1371/journal.pone.0013513 (PMC2958123; doi:10.1371/journal.pone.0013513)

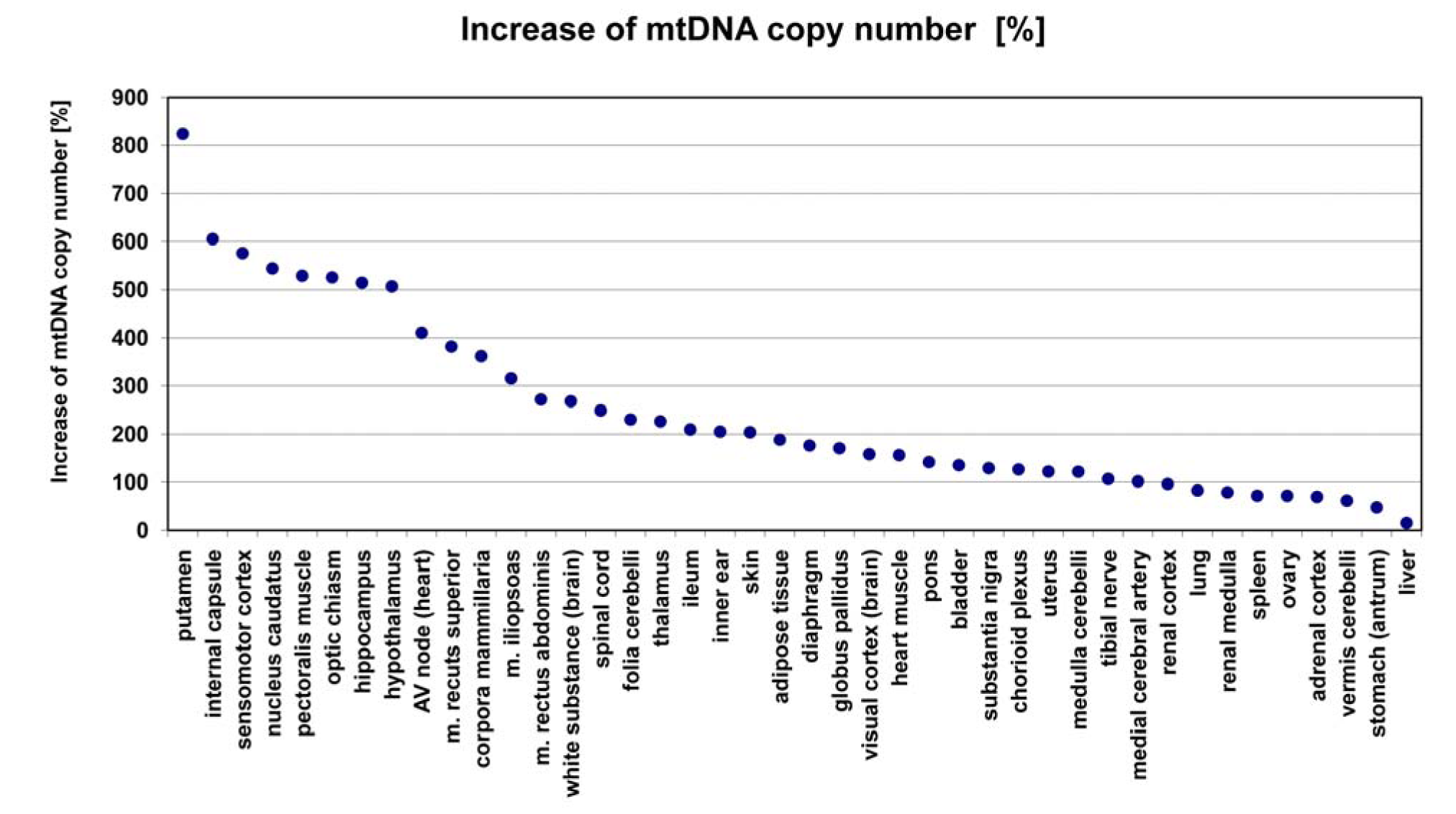

Supplement: Figure S1 — Increase [in %] of the mtDNA copy numbers in the patient in relation to the mean of the four controls. The tissues mainly affected by the MERRF-syndrome cluster on the left side of the chart. (0.65 MB TIF) [file pone.0013513.s001.tif]

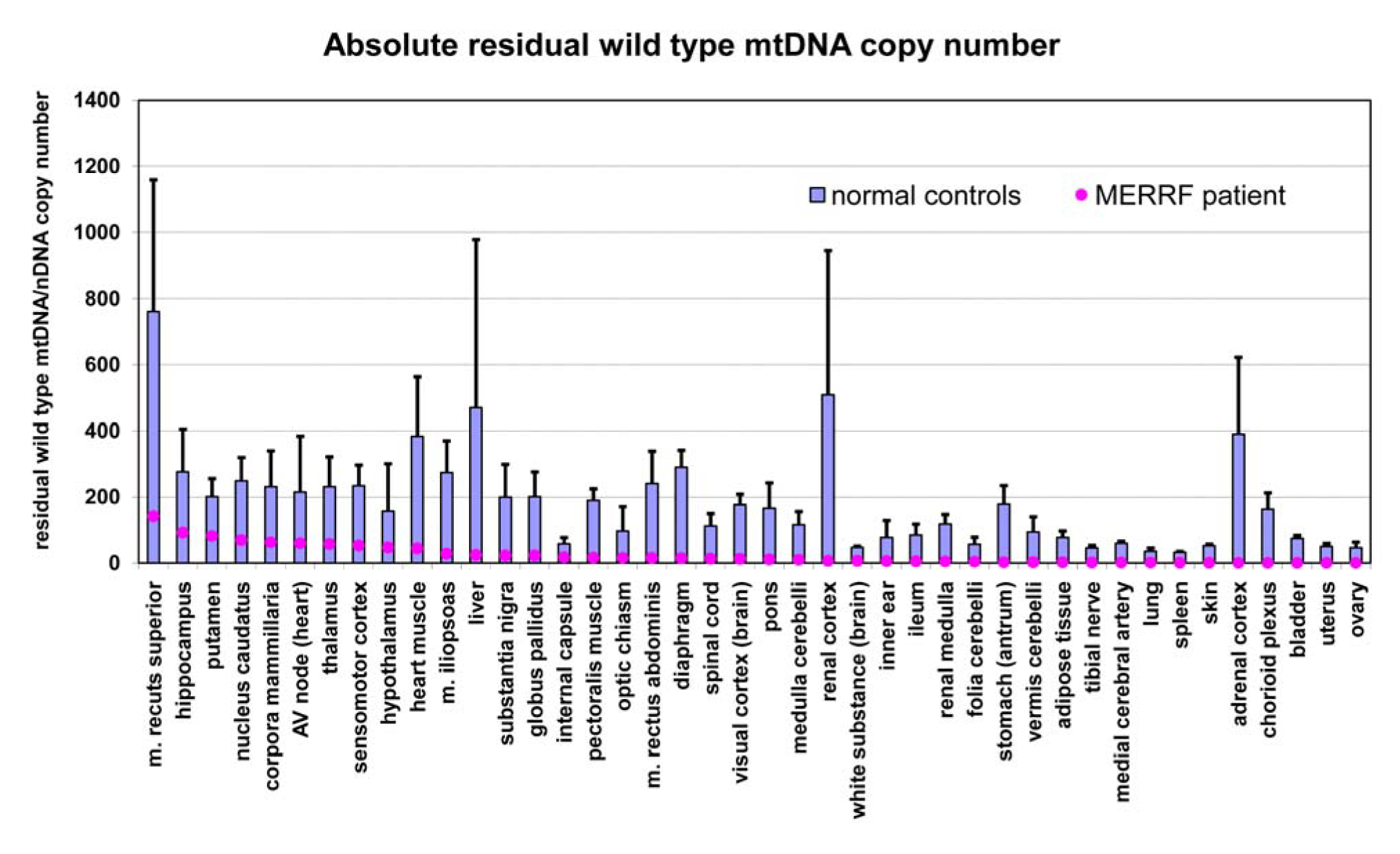

Supplement: Figure S2 — Absolute numbers of residual wildtype mtDNA molecules per cell in the patient tissues (magenta dots). The blue bars depict the mean and the whiskers the standard deviation of the mtDNA copy numbers in the four controls. (1.01 MB TIF) [file pone.0013513.s002.tif]

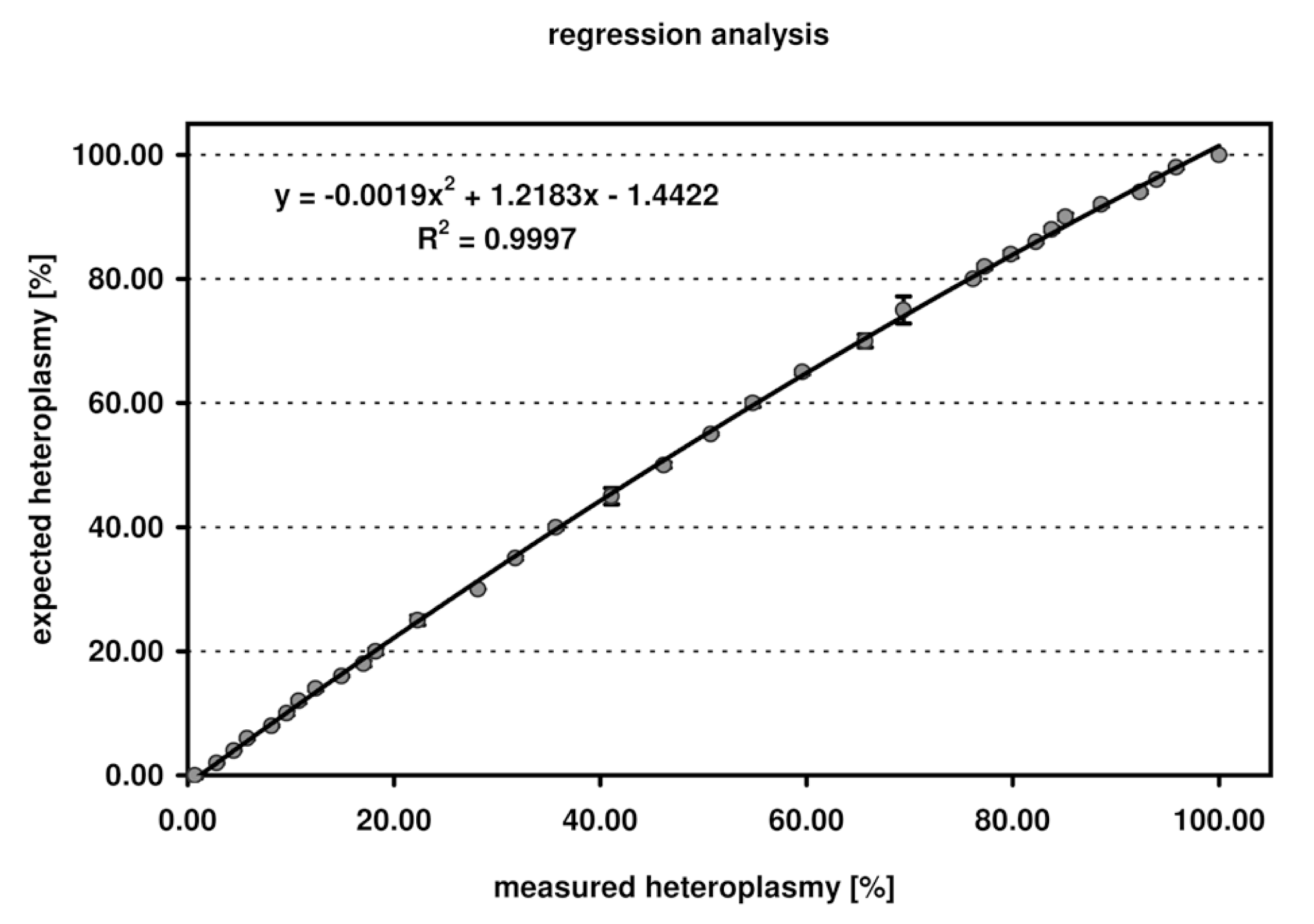

Supplement: Figure S3 — Calibration curve for the Pyrosequencing assay. Measurements from the assay were plotted against the expected degrees of heteroplasmy from known mixtures between plasmid preparations containing the wildtype and mutant DNA sequence. All measurements were performed in triplicate and the standard deviation is indicated by whiskers. Due to the high precision of the method with small standard deviations, not all the whiskers can be seen. The regression line forms a smooth curve with the above mentioned equation and a very good approximation of the measurements. All raw measurements of the samples were normalized to the regression curve. (0.33 MB TIF) [file pone.0013513.s003.tif]

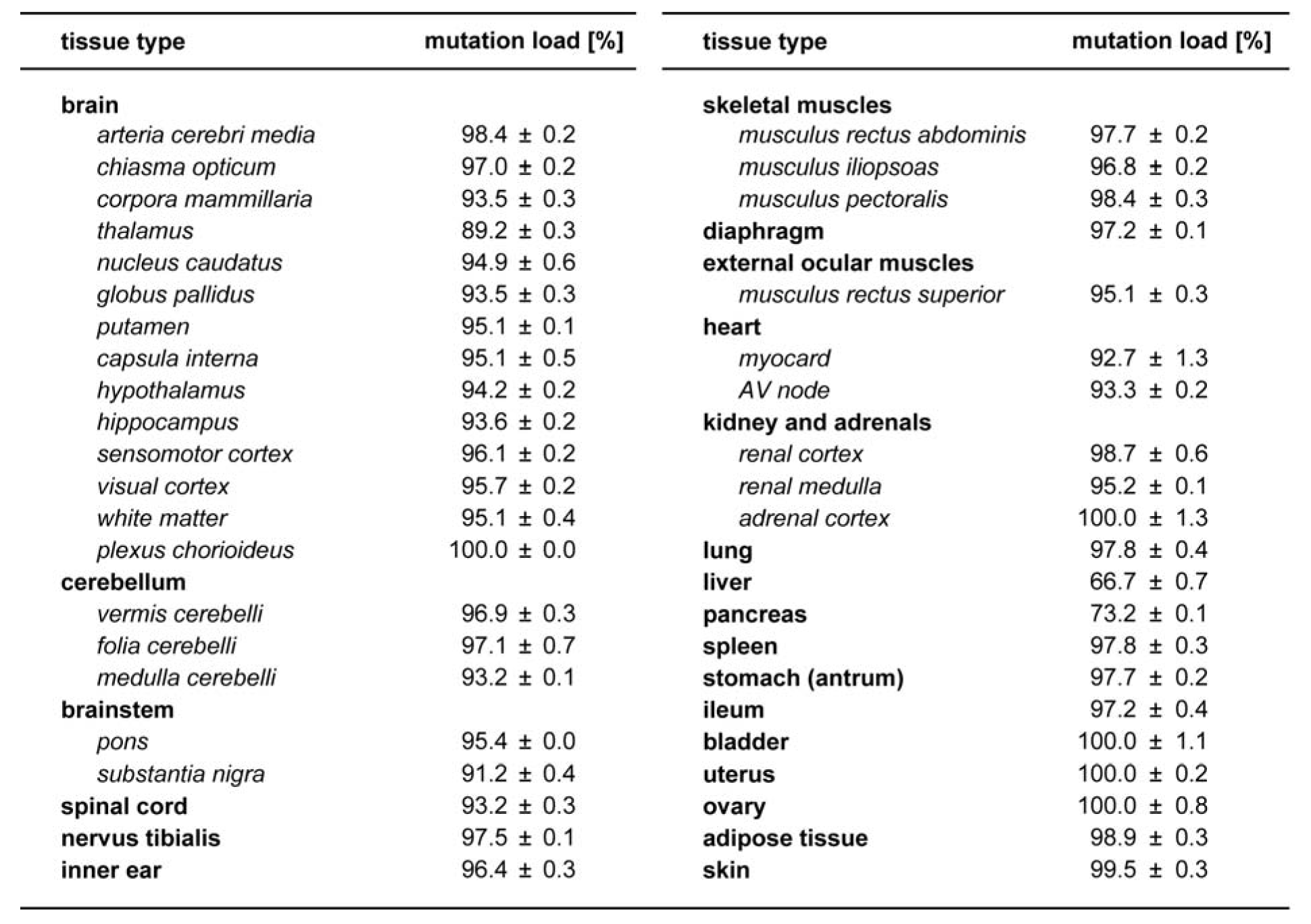

Supplement: Table S1 — Mutation loads ( = degrees of heteroplasmy in [%]) of all the organs as measured by the Pyrosequencing assay. The numbers indicate the average of three measurements ± standard deviation. (0.95 MB TIF) [file pone.0013513.s004.tif]

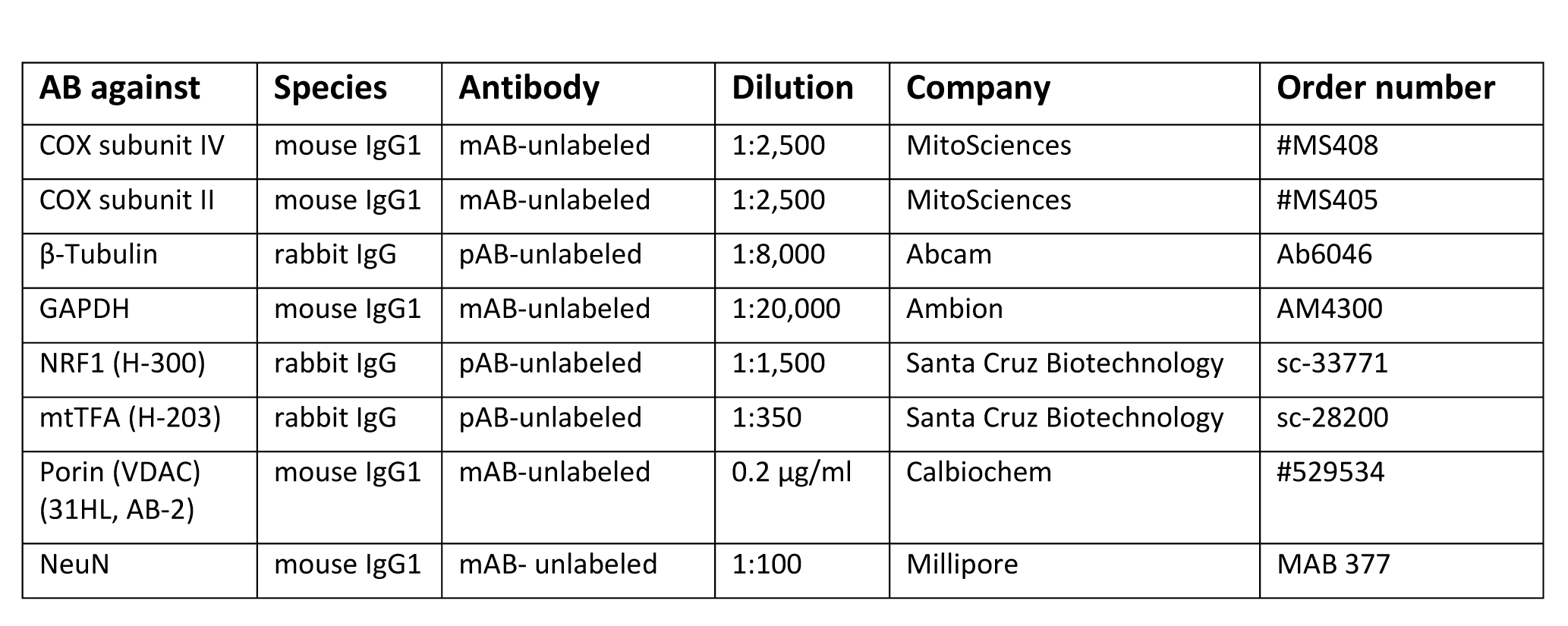

Supplement: Table S2 — Primary antibodies and their dilutions used for Western blot and immunohistochemistry. (0.11 MB TIF) [file pone.0013513.s005.tif]
